# Translating health research evidence into policy and practice in Uganda

**DOI:** 10.1186/1475-2875-12-274

**Published:** 2013-08-05

**Authors:** Anthony K Mbonye, Pascal Magnussen

**Affiliations:** 1 Associate Professor, School of Public Health, College of Health Sciences-Makerere University & Commissioner Health Services, Ministry of Health, Box 7272, Kampala, Uganda; 2 University of Copenhagen, Centre for Medical Parasitology, Copenhagen, Denmark

**Keywords:** Policy, Health research evidence, Health system reforms, Collaboration, Uganda

## Abstract

**Background:**

Uganda experiences a high disease burden of malaria, infectious and non-communicable diseases. Recent data shows that malaria is the leading cause of morbidity and mortality among all age groups, while HIV prevalence is on the increase and there is re-emergence of viral haemorrhagic fevers and cholera epidemics. In order to respond to the above situation, a team of researchers, policy makers, civil society and the media was formed in order to build a collaboration that would help in discussing appropriate strategies to mitigate the high disease burden in Uganda.

**Methods:**

A preparatory secretariat composed of individuals from Ministry of Health, Malaria Research Centre and School of Public Health was formed. The secretariat identified researchers, key resource persons to guide the workshops and the format for presentation. The criteria for selection of the research topics were: National public health importance and had been published in peer-reviewed journals. The presentations were structured as follows: research questions, hypotheses, methodology, major findings and policy implications. The secretariat compiled all the proceedings of the workshops including attendance, address of participants including telephone and email contacts. During the last workshop, an evaluation was conducted to assess the impact of the workshops.

**Results:**

Four workshops were held between 2006 and 2009. A total of 322 participants attended of whom mid-level policy makers, researchers and the media were consistently high. The workshops generated a lot of interest that lead to presentation and discussion of nationally relevant health research results. The workshops had an impact on the participants’ skills in writing policy briefs, participating in the policy review process and entering into dialogue with policy makers.

**Conclusion:**

The following lessons have been learned: getting health research into policy is feasible but requires few self-motivated individuals to act as catalysts. Adequate funding and a stable internet are necessary to support the process. Mid-level policy makers and programme managers had interest in this initiative and are likely sustain it as they move to senior positions in policy making.

## Background

Uganda experiences a high disease burden of malaria, infectious and non-communicable diseases. Recent data shows that malaria is the leading cause of morbidity and mortality among all age groups. Therefore, there is need to constantly review existing health policies and developing new ones should be prioritized. It is now evident that Uganda will not achieve the Millennium Development Goals (MDGs). Recent data shows that the prevalence of malaria, diarrhea diseases, and upper respiratory tract infections is high [1,2]. Similarly, the prevalence HIV is on the increase [1] and there is re-emergence of epidemics of viral haemorrhagic fevers and cholera [3]. Data from the Demographic and Health Surveys show that impact indicators on health have stagnated over a long period. Maternal mortality ratio was 435/100,000 live births in 2006 and has stagnated at 438/100,000 live births in 2011, while infant mortality rate has declined slightly to 54/1000 live births [1].

Because of the poor state of health mentioned above, Uganda has implemented health sector reforms like restructuring of the Ministry of Health, health care financing, sector-wide approaches and decentralization of health services [2,4]. Other policies like the Malaria Control Policy, The Road Map to reduce maternal morbidity and mortality, the child survival strategy, the HIV/AIDs prevention and Control Policy are in place and aimed to improve access and utilization of health services. However, it seems these polices have not been effectively implemented [5]. Several constraints that hinder delivery of health services in developing countries including Uganda have been documented and include inadequate financing, low staff levels, socio-economic constraints, non-friendly services and poorly designed policies [6-10].

Reviewing and implementing policies is complicated and takes a long time; and this may lead to negative consequences on health outcomes. For example, chloroquine resistance was reported in Uganda in 1994; but it took several years to revise the malaria treatment policy to a combination of chloroquine and sulphadoxine-pyrimethamine [11]. Similarly, drug resistance to sulphadoxine-pyrimethamine was reported in 2000, but it took five years to revise the policy to artemisinin combination therapy (ACT) [12-14]. More recently, it has been demonstrated that community-based distribution of intermittent preventive treatment (IPTp) for prevention of malaria in pregnancy increases access leading to reduced prevalence of anaemia and low birth weight [13]. However, the relevant policy has not been reviewed. Similarly, Uganda is one of the countries that pioneered community-based distribution of the medroxy-progesterone acetate contraceptive [15]. The study showed that community health workers can safely give injectable contraceptives, but, while several African countries have revised their policies to scale up this intervention, the relevant policy has only recently been revised in Uganda [14,16,17].

In Uganda, implementation of health services is decentralized [2]. The design and initiation of health policies is a function of the central level; while the lower levels, district and sub-district health authorities implement the policies. At the design and initiation of policies, there are no wide consultations with stakeholders and this may affect acceptability and impede implementation of policies.

The current Health Sector Strategic Plan (HSSPIII) has indicators to monitor health sector performance [5]. However, data from the periodic monitoring and evaluation (M&E) exercises do not lead to policy discussions and reviews; yet this is one of the ways to evaluate the effectiveness of policies. In the current HSSPIII set-up, there is an M&E -research technical working group that is supposed to provide oversight on research activities [5]. However, this working group has no representation from local health authorities, civil society, the media and academia.

The Uganda National Health Research Organization (UNHRO) has been established to coordinate research. UNRHO has the potential to bring research evidence to the attention of policy makers and facilitate regular meetings between researchers and policy makers. Although the Ministry of Health (MOH) has the mandate to initiate and develop polices to improve health status in Uganda, the structures responsible for this are sector working groups clustered around diseases and support functions of the ministry with no framework to initiate, discuss or review policies.

Given the above scenario, it was conceived that researchers and policy makers in Uganda start a dialogue on how to improve policy making and implementation. This article presents results based on four annual research-to-policy workshops. The four-year experience and lessons learned are presented.

## Methods

### Conceptualization of research to policy workshops

In 2006, interested researchers at the MOH and the University of Copenhagen got concerned about the high disease burden in Uganda and how to make use of good quality and relevant research available to inform policy and practice. Several discussions were held and concluded that a dialogue between researchers, policy makers and programme managers had to start. Part of the discussion was why many policy recommendations were not implemented. Discussions also aimed at identifying stakeholders important in research to policy and practice. The following institutions were identified: School of Public Health Makerere, College of Health Sciences, Makerere University; Uganda National Health Research Organization (UNHRO), Malaria Research Centre, the Media, Civil Society, District Health Authorities and MoH policy makers and programme managers (the Director General, Commissioners, Assistant Commissioners, Vector Control Division, AIDS Control Programme, Malaria Control Programme, Child Health Division, Non-Communicable Diseases, Communicable Diseases Division and Reproductive Health).

### Implementation of research to policy workshops

Initially it was conceived that a workshop presenting existing study results that could have impact on policy be organized; and in that workshop discussions be held on the direction future dialogues would take. A preparatory secretariat composed of key individuals from Ministry of Health, Malaria Research Centre and School of Public Health was formed. The secretariat identified researchers and topics for discussion and the format for presentation. The criteria for selection of the research topics were: National public health importance and had been published in peer reviewed journals. The presentations were structured as follows: research questions, hypotheses, methodology, major findings and policy implications. The secretariat compiled all the proceedings of the workshops including attendance, addresses of participants including telephone and email contacts. Research papers presented were also shared among participants. Four workshops were carried out between 2006 and 2009. The first workshop discussed research evidence and identified policy implications. At the end of the workshop, a database of researchers and policy makers was created. The second and third workshops also discussed research evidence and identified policy implications in addition to hands-on skills on writing policy briefs. While the fourth facilitated researchers to present policy briefs.

### Evaluation of the impact of workshops

Four workshop reports were reviewed and the following data extracted: number of participants in each workshop, home institution for each participant and the research presented at the workshop or submitted. During the fourth workshop, a semi-structured questionnaire was developed and administered to participants to capture data on: the individual characteristics like qualifications, current research activities and how they perceived they had gained from the current and previous workshops.

### Data analyses

Data from the semi-structured questionnaire was entered and analyzed using Stata Version 11.0 and frequencies on individual variables computed. Qualitative data extracted from reports were manually analyzed on themes of interest like benefits from the workshops, interactions between researchers and policy makers, sustainability and suggestions on future workshops.

## Results

A total of 322 participants attended the workshops over the four-year period. The number of participants increased over time. Analyses show that 83.4% of the participants consistently attended all the four workshops. Overall, there was a 46% increase in the number of participants since the workshops were conceived (Table 1). The three main groups of participants that attended consistently were: researchers, policy makers and the media (Table 1).

**Table 1:** Category of participants

**Participants**	**2006**	**2007**	**2008**	**2009**
	**Frequency (%)**	**Frequency (%)**	**Frequency (%)**	**Frequency (%)**
Policy makers	12 (17.7)	13 (18.8)	17 (19.3)	24 (24.5)
Researchers	15 (22.1)	23 (31.5)	25 (28.4)	26 (26.5)
District officials	6 (8.8)	5 (6.9)	8 (9.1)	3 (3.1)
Civil society	4 (5.9)	5 (6.9)	6 (6.8)	9 (9.2)
Media	15 (22.1)	12 (16.4)	17 (19.3)	18 (18.4)
Programme managers	8 (11.7)	6 (8.2)	10 (11.4)	8 (8.2)
Graduate students	8 (11.7)	9 (12.3)	5 (5.7)	10 (10.2)

The research areas covered varied from workshop to workshop depending on the availability of the researchers and the published data available. Over the four years, the topics covered ranged from , malaria, neglected tropical diseases, infectious diseases, health economics, obstetrics, health systems, policy development, and public health.

An evaluation of the impact of the four workshops was carried out. A proportion of participants said they had gained scientific knowledge; others had opportunity for knowing new research areas and appropriate methodology, while others had gained knowledge on the importance of research, evidence based decision making and networking with other researchers (Table 2).

**Table 2:** Impact of research to policy workshops, 2009

**Reported impact**	**Number of**
	**participants**
	**Frequency (%)**
Gained scientific knowledge	20 (20.4)
Networking with researchers	8 (8.2)
Written policy briefs and reports for dissemination	6 (6.1)
Reviewed policy guidelines	1 (1.0)
Written new articles	2 (2.0)
Developed a radio program	3 (3.1)
Written research proposal	3 (3.1)
Written research articles for publication	5 (5.1)
Got to know new areas for research and methodologies	11 (11.2)
Understood how to write policy briefs	1 (1.0)
Motivated and developed interest in doing research	7 (7.1)
Appreciated the importance of research and evidence based decision making	12 (12.2)
Improved skills and knowledge in advocacy and communication with facts	2 (2.0)
Gained experience in organizing workshops	6 (6.1)
Improved knowledge in evaluating research methodology and practice	1 (1.0)
Did not benefit so much due to lack of follow-up	1 (1.0)
Timely for the revision of the national health policy and HSSPII	5 (5.1)
Gained knowledge on the policy development process	4 (4.1)
**Total**	**98**

Of all 98 participants who attended the last work shop, 20 (20.4%) had gained scientific knowledge, 6 (6.2%) had written policy briefs, 12 (12.2%) had appreciated the importance of research and evidence based decision making, and 3 (3.1%) had since developed research proposals and were looking for funding (Table 2). It was reported that several policies had been reviewed and these included: Reproductive Health Policy Guidelines (revised to allow community based provision of Injectable Depo-provera); Integrated Community Case management of childhood illnesses, Village Health team strategy, Malaria Control Policy, Medical male circumcision, Infant and Young child feeding, Prevention of Mother to Child Transmission (PMTCT) and the Road Map for improving maternal and newborn Care.

## Discussion

Over the four-year period, several successes were achieved. Primarily the number of relevant stakeholders that participated increased from workshop to workshop. Secondly, the interest this initiative generated among the participants lead to presentation and discussion of nationally relevant health research results, which may never have happened. Thirdly, the workshops have had an impact on the participant’s skills in writing policy briefs, participating in the policy review process and entering in dialogue with policy makers. The interesting part of this initiative was the high involvement of the media and their interest was to report important research findings. How this impacted on policy makers, researchers and the media personnel has not been established and should be assessed in future.

The above was achieved partly because the workshops were conceived by researchers close to the policy making process with a background of health research and being concerned how the research impacted on policy and practice and eventually benefited the population. Through personal contacts and dialogue with colleagues, researchers and policy makers have increasingly got interested in this area.

The other important factor for the success was institutional collaboration between the School of Public Health, Makerere University and the involvement of the interim Uganda National Health Research Organisation (UNHRO). The involvement of these two institutions was sought early as the former had the capacity to generate research evidence or collate the results of previous research conducted; while the latter would sustain this initiative. In fact, future policy workshops will be spearheaded by UNHRO.

Awareness creation among policy makers about the importance of a dialogue between researchers and policy makers as a way to improve programmes through adaptation of relevant research findings in the policy change process was seen as crucial. It was also mutually agreed that there was a need to strengthen the capacity of UNHRO by recruiting personnel and provide funding to carry out its mandate to coordinating the various research initiatives and organizations to help policy makers identify programme constraints and allocate adequate funding to research. One observation was that there were few senior policy makers (Permanent Secretary, Director General of Health Services, Commissioners and Assistant Commissioners) in these workshops. They came briefly either at the opening or closing of the workshops. A strategy to involve mid-level policy makers and managers (Principal Medical officers, Program Managers, and Senior Medical Officers) in research generation and policy development process is likely to achieve sustainable results as these managers are likely move to senior policy making positions.

Other initiatives exist at a country level spearheaded by the College of Health Sciences-Makerere University to address the issue of research to policy and knowledge translation. The East African Community established the Regional East African Community Health (REACH) Policy Initiative within the East African Health Research Commission (EAHRC), a regional, intergovernmental organ charged with overseeing health research and translating research findings into policy and practice [18]. UNHRO is the National Focal Point for the EAHRC and hence for the REACH Policy Initiative. The College of Health Sciences, Makerere University is collaborating with UNHRO under the Supporting Use of Research Evidence (SURE) for policy project which builds on and supports the REACH Policy Initiative and the African Evidence-Informed Policy [19]. It is recommended that all initiatives at a country level that aim to bring research evidence to policy should come together to strengthen this initiative.

In the recent past, several polices have been reviewed at the MoH; and it is believed that the workshops may have contributed to this. A review of the policies shows that some of the literature referenced was presented at the workshops. Several players contributed to the development and review of policies and therefore we are conscious of that fact as we interpret these findings.

In convening the workshops, the following constraints were experienced: lack of fast and stable internet connection to reach researchers and policy makers to share vital research material; the busy schedule of top policy makers leave no time to stay a whole day in a workshop to learn and appreciate the use of research evidence for policy making; inadequate funding to support a large number of participants; identifying self-motivated catalysts to advocate for this initiative and identifying credible research evidence (robust study design, generalizability of findings). If this initiative is to be sustained, these constraints have to be addressed.

During the last workshop it was recommended that future workshops be spearheaded by UNHRO. Prior to the workshops, programme managers should identify policy issues or constraints for which data requirements would be discussed. In addition, a framework for data collection and policy review would be agreed on. This is likely to lead to a quick review of policies in Uganda.

The recent passing of the UNHRO bill by Parliament should lead to creation of a vibrant UNHRO with personnel and adequate funding and hopefully UNHRO may then be able to spearhead a process of transforming relevant research findings into policy and practice in order to address the high burden of disease in Uganda.

The following lessons have been learned overtime: getting health research into policy is feasible but requires patience and persistence of self-motivated individuals. Adequate funding and a stable internet are necessary to support the process. Mid-level policy makers and program managers had interest in this initiative and are likely sustain it as they move to senior positions in policy making. The media was highly involved in this initiative and are important for advocacy but also to call partners to action. The collaboration between Northern and Southern institutions was key to this initiative initially at the conceptualisation and the implementation phase. Such collaboration not only built capacity but facilitated learning from each other.

## Competing interests

The authors declare that they have no competing interests.

## Authors’ contributions

AKM and PM conceptualized the research into policy workshops, and participated in the workshops, data analyses and preparation of the manuscript. Both authors read and approved the manuscript.
